# Iron Homeostasis in the CNS: An Overview of the Pathological Consequences of Iron Metabolism Disruption

**DOI:** 10.3390/ijms23094490

**Published:** 2022-04-19

**Authors:** Christina A. Porras, Tracey A. Rouault

**Affiliations:** Eunice Kennedy Shriver National Institute of Child Health and Development, Bethesda, MD 20892, USA; christina.porras@nih.gov

**Keywords:** iron metabolism, iron homeostasis, iron regulatory proteins, neurodegeneration, iron accumulation, iron deficiency

## Abstract

Iron homeostasis disruption has increasingly been implicated in various neurological disorders. In this review, we present an overview of our current understanding of iron metabolism in the central nervous system. We examine the consequences of both iron accumulation and deficiency in various disease contexts including neurodegenerative, neurodevelopmental, and neuropsychological disorders. The history of animal models of iron metabolism misregulation is also discussed followed by a comparison of three patients with a newly discovered neurodegenerative disorder caused by mutations in iron regulatory protein 2.

## 1. Iron Metabolism

As the most abundant trace element in the human body, iron is vital to many different facets of biology. Iron physiologically can switch between two oxidation states: ferric (Fe^3+^) and ferrous (Fe^2+^) using very little energy. This ability allows iron to act as both an electron donor and an electron acceptor in redox reactions. Iron is, therefore, essential for the proper functioning of many proteins with roles in DNA synthesis, mitochondrial respiration, hemoglobin synthesis, and the formation of iron–sulfur clusters [1].

In aerobic conditions at physiological pH, the highly insoluble ferric iron is the predominant state of iron. Iron transport systems, however, only take in ferrous iron, which is unstable and rapidly oxidizes to ferric iron. To deal with this problem, the ferrireductase duodenal cytochrome B (DcytB) reduces dietary iron in the duodenum [2]. Ferrous iron is then absorbed into the enterocyte by divalent metal transporter 1 (DMT1), a membrane iron transporter [3]. Internalized ferrous iron can remain inside the cell either as part of the labile iron pool (LIP) or bound to the iron storage protein ferritin and sequestered. Ferrous iron can be exported through the basolateral iron exporter ferroportin 1 (FPN1) [4,5,6]. The exported ferrous iron is oxidized back to ferric iron by hephaestin, a ferroxidase, and then binds to transferrin (Tf) [7]. This diferric transferrin complex (diferric-Tf or holo-transferrin) circulates through the blood stream acting as the major pool of iron for most tissues [1]. Diferric-Tf binds to transferrin receptor 1 (TfR1), which is then internalized into an endosome, where acidification releases ferric iron from diferric-Tf yielding apo-transferrin. Ferric iron is then reduced to ferrous iron by the endosomal ferrireductase called six-transmembrane epithelial antigen of the prostate 3 (Steap3) [8]. The free ferrous iron is then transported into the cytosol by DMT1, where it can then be incorporated into iron proteins with the help of chaperone proteins or enter the mitochondria through the mitochondrial iron importers mitoferrin 1 or mitoferrin 2 [9,10].

## 2. Iron Regulatory Proteins: Expression, Regulation, and Function

Two proteins are responsible for the maintenance of cellular iron homeostasis, iron regulatory protein 1 and iron regulatory protein 2. IRP1 and IRP2 share 58% homology, and they concordantly regulate the expression of iron metabolism proteins. These regulatory proteins are expressed ubiquitously, but in particular cells and tissues, one may have a higher expression level than the other and, thus, be relatively more dominant. IRP1 is the more relatively abundant protein in kidney, liver, and brown fat, whereas the expression of IRP2 relative to IRP1 is high in the central nervous system (CNS) including the forebrain, cerebellum, substantia nigra, and hippocampal regions of the brain [1,11].

Though both of these proteins become active during iron-deficient conditions, the mechanism of their activation and regulation differs [12]. IRP1 switches between two functions based on whether it ligates an [4Fe–4S] cluster. In IRP1′s holo-form, IRP1 acts as a cytosolic aconitase, interconverting citrate and isocitrate. Most of the cellular IRP1 ligates an iron–sulfur cluster when cellular iron concentrations are high. During low-iron conditions, however, iron–sulfur cluster biogenesis is compromised and fails to generate the [4Fe–4S] cluster for IRP1 to ligate. In its apo-form, IRP1 undergoes a conformational change to become an RNA-binding protein. IRP2 does not bind an iron–sulfur cluster; it undergoes proteasomal degradation in response to high-iron conditions. Degradation is initiated by an E3 ubiquitin ligase complex containing the F-box protein, FBXL5. FBXL5 is activated when iron and oxygen bind to its hemerythrin domain. FBXL5 also contains an oxygen-sensitive iron–sulfur cluster, which upon reduction binds to IRP2. As a result, the activity and protein levels of IRP2 increase during iron-deficient and hypoxic conditions due to inactivation or degradation of FBXL5 [13,14,15,16,17].

The result of these regulation mechanisms is that during low intracellular iron concentrations IRPs bind to RNA stem-loop structures called iron response elements (IREs) that are present on the transcripts of iron metabolism proteins [18]. The effect of this interaction depends on whether the IRE is present on the 5′UTR or 3′UTR of the transcript. If the IRE is present on the 5′UTR, binding of IRP1/2 prevents the initiation of translation and downregulates the iron metabolism protein. Inhibition of translation by IRPs occurs for iron metabolism proteins necessary for iron storage and export including ferritin; FPN1; mitochondrial aconitase (ACO2), necessary for energy production; erythroid 5-aminolevulinic acid synthase (eALAS), the first protein in heme synthesis; and hypoxia inducible factor 2α (HIF2α), which senses hypoxia and erythropoiesis [4,5,19,20,21,22]. Conversely, IRP binding to the 3′UTR protects the transcript from degradation and, thereby, increases the synthesis of the iron metabolism proteins such as TfR1 and DMT1 [23,24]. The ability of IRP binding to coordinate the upregulation of iron import proteins and downregulation of storage and export proteins leads to an increase in intracellular iron concentrations in response to iron-deficient conditions and the maintenance of iron homeostasis [1,25].

## 3. Iron in the CNS

The entry of iron into the CNS is made more complicated by the blood–brain barrier (BBB) and the blood–CSF barrier, which separate the CNS from the systemic circulation of iron [26]. Holo-transferrin binds to TfR1 on endothelial cells on the luminal side of the BBB. FPN1 may then alone or with the aid of a membrane-bound form of the ferroxidase ceruloplasmin (Cp) present on astrocytic foot processes export iron across the abluminal membrane into the interstitial fluid [27,28]. Another pathway for iron to enter the CNS is through cells of the choroid plexus, which also synthesize and secrete transferrin. Once in the interstitial fluid or CSF, iron binds to locally synthesized transferrin. The resulting holo-transferrin binds to TfR1 to deliver iron to the various cells of the CNS [26,28,29].

Most cell types express all the proteins necessary for iron metabolism. In the CNS, however, there is some specialization due to the varying amounts of iron protein expression. Oligodendrocytes are the main source of transferrin. Microglia highly express the storage protein, ferritin, whereas neurons highly express TfR1 to import the necessary amount of iron for active metabolism [30]. The ferroxidases ceruloplasmin and hephaestin share 50% homology and are expressed throughout the CNS, but ceruloplasmin is mainly synthesized by astrocytes, where alternative splicing leads to the attachment of a glycosyl phosphatidylinositol (GPI) that links ceruloplasmin to the membrane of the astrocyte. Outside the CNS, most ceruloplasmin is secreted into circulation. Hephaestin is a membrane-linked ferroxidase that is mainly synthesized by oligodendrocytes. These differences in expression of iron metabolism proteins have led to the hypothesis that each of the CNS cell types has variable requirements for iron, and therefore, they distribute and store iron according to their own needs and functions [7,28,30,31,32].

## 4. Neurological Disorders Associated with Iron Accumulation

Because of the many different proteins that utilize iron, maintenance of iron homeostasis is critical. This is especially true in the brain, where it is used for myelination, neurotransmitter synthesis, oxygen transport, and in the mitochondrial respiratory chain to generate enough energy to meet the high metabolic demand of neurons [33]. It is known that the brain acts as an iron repository and that ferritin iron gradually accumulates in the brain over time before stabilizing in adulthood. Accumulation of iron-loaded ferritin is prominent in the globus pallidus, red nucleus, dentate nucleus, and the substantia nigra, though why these regions of the brain store high amounts of iron is unknown [34]. One clue may be that during periods of iron deficiency capable of depleting the liver’s stores of iron, brain iron concentrations remain constant. Thus, the brain’s accumulation of iron in specific regions may represent an effort to ensure a stable iron supply for the brain [28,32]. 

### 4.1. Iron Accumulation in Neurodegenerative Diseases

Brain iron accumulation is also associated with several neurodegenerative diseases, though in most cases, it is unknown whether iron accumulation causes or results from these neurodegenerative diseases. The ability of iron to catalyze redox reactions also causes the generation of reactive oxygen species through Fenton chemistry, which can generate hydroxyl radicals, which can damage DNA, RNA, proteins, and lipids [35,36]. MRI techniques have shown that iron accumulates in the brains of Parkinson’s patients, particularly in the substantia nigra. Preclinical studies have demonstrated that iron chelators can cross the BBB and attenuate the loss of dopaminergic neurons [37]. Deferiprone therapy in a phase 2 clinical trial showed a reduction in iron content in the dentate and caudate nuclei of Parkinson’s patients, but only three patients had a reduction in the substantia nigra, and no changes were observed in the putamen or globus pallidus [38]. FairPark II is a randomized, phase 2 trial for conservative iron chelation therapy in PD, which is estimated to be completed in 2022 and will offer additional insight into the efficacy of iron chelators in PD [39,40]. MRI lacks sufficient resolution to determine which cell types accumulate iron, so the observed iron accumulation may not correspond to iron overload in neurons, as has been commonly assumed [28]. In fact, MRI has also shown iron accumulation in the motor cortex of ALS patients. A postmortem pathological study revealed that, in these iron-rich areas of the brain, motor neurons had been replaced with iron-rich activated microglia [41]. It is unclear whether these microglia are accumulating iron, activating, and then infiltrating into the region or whether they are “cleaning up” the iron left behind when iron-overloaded neurons die. This is an important distinction because iron chelators may not be helpful after neural death has occurred [28].

In 1998, IRP2 was associated with intraneuronal lesions including neurofibrillary tangles, senile plaque neurites, and neuropil threads in postmortem brain tissue from patients with Alzheimer’s disease [42]. A decade later, a decrease in brain copper in the parietal cortex, hippocampus, ventral striatum, thalamus, hypothalamus, and whole brain was reported in six-week-old Irp2^−/−^ mice with also an increase in amyloid-b protein precursor (AbPP) in the hippocampus [43]. An IRE has also been suggested to function on the 5′-UTR of amyloid precursor protein (APP) mRNA, which is bound strongly by IRP1 but not IRP2 [44]. Knockdown of IRP1 in SH-SY5Y cells increased APP expression 17-fold due to removal of the translational block of IRP1 [45]. High concentrations of iron can then promote the amyloidosis pathway of APP cleavage resulting in generation of Ab_1–42_ fragments, which, in combination with Fe^2+^, can cause oxidative damage [46,47,48].

### 4.2. Ferroptosis and Iron Metabolism

In 2012, the term ferroptosis was coined to describe a form of regulated cell death distinct from apoptosis, necrosis, and autophagy that is caused by the iron-dependent accumulation of lipid hydroperoxides [49,50]. Though ferroptosis may be important for tumor suppression, little is known about its function outside of pathological contexts [51]. The importance of several iron metabolism proteins in the execution of ferroptosis has been demonstrated. These include transferrin and transferrin receptor, IRP2, and its regulator, FBXL5, and ferritin by way of ferritinophagy, i.e., the selective autophagy of ferritin by NCOA4 [49,52,53,54].

Glutathione peroxidase 4 (GPX4) inhibits ferroptosis by repairing oxidative damage to lipids. Ablation of this protein in the neurons of mice results in paralysis and degeneration of motor neurons [55]. Conditional knockout of GPX4 in forebrain neurons caused significant deficits in spatial learning and memory and hippocampal neurodegeneration, which was amplified when the mice were fed a diet deficient in the anti-ferroptosis antioxidant vitamin E [56]. In a rat model of intracerebral hemorrhage (ICH), overexpression of GPX4 was protective against brain injury [55]. Young tau-knockout mice are protected from reperfusion injury following middle cerebral artery occlusion (MCAO). Brain iron accumulation negates this benefit in tau-knockout mice at 12 months of age, but protection from MCAO reperfusion injury can be restored following ceruloplasmin treatment or ferroptosis inhibitors [57]. Treatment with ferrostatin-1 to inhibit ferroptosis in the hippocampus after kainic acid (KA)–induced temporal lobe epilepsy (TLE) decreased neuron cell death, cognitive impairment, and iron accumulation (but not the occurrence of spontaneous seizures) by restoring GPX4 expression [58]. Inhibition of ferroptosis may, therefore, offer a therapeutic method to reduce nerve injury after epileptic seizures [59]. More generally, ferroptosis may be an important aspect of many neurological diseases characterized by iron accumulation.

### 4.3. Neurodegeneration with Brain Iron Accumulation Diseases

Neurodegeneration with brain iron accumulation (NBIA) diseases are a group of rare diseases caused by known germline mutations that can present with movement dysfunction, abnormal gait, spasticity, as well as motor and cognitive dysfunction. Iron accumulation typically occurs in the globus pallidus, but in a few of the diseases, iron also accumulates in the cerebellum and substantia nigra [60]. Not all the NBIA diseases have a direct link to iron metabolism. One that does is aceruloplasminemia, which is caused by mutations in ceruloplasmin (CP). In this disease, iron overload is observed in the basal ganglia along with the loss of neurons and accumulation of globular astrocytic remnants [61]. The cause of this neuron loss is unclear. Astrocytic ceruloplasmin is required for iron export out of astrocytes. It is, therefore, possible that loss of ceruloplasmin results in an increase in non-transferrin-bound uptake of iron. Unable to export this iron, astrocytes would then become iron overloaded. Astrocytes in this situation may “hoard” iron, causing nearby neurons to become iron deficient. Neuronal iron deficiency along with the added toxic insults from adjacent dying astrocytes could cause the observed loss of neurons in the basal ganglia [28].

A second NBIA disease with a direct link to iron metabolism is neuroferritinopathy (NF), which is caused by dominantly inherited mutations in L-ferritin (FTL) [62]. Iron accumulation is seen in the basal ganglia, and the disorder is characterized by extrapyramidal movement disorders, dystonia, parkinsonism, and dysarthria [63]. Mutations in exon 4 in the FTL1 sequence affect the length and sequence of the C terminus peptide and in a dominant-negative manner impair the ability of ferritin to store iron [64]. Recently, a group developed an NF model using iPSC-derived neuronal precursor cells (NPCs) and neurons derived from the fibroblasts of NF patients. NF fibroblasts, neural progenitors, and neurons showed an increase in free iron and ferritin/iron aggregates, ROS and lipid peroxidation, senescence, and ferroptotic cell death [64]. Together, these two NBIA disorders may offer opportunities for studying the effects of iron metabolism misregulation in the future with implications for other neurodegenerative diseases associated with iron accumulation.

## 5. Iron Homeostasis and Behavior

### 5.1. Cognitive Effects of Iron Deficiency

The relationship between iron homeostasis and behavior has most extensively been studied in terms of iron deficiency particularly iron deficiency’s effect on brain development during pregnancy and early childhood. Iron deficiency is most prevalent among pregnant women, infants, and young children, though it is the most common micronutrient deficiency overall, usually as a result of inadequate dietary intake or insufficient absorption [65]. Iron deficiency (ID), where the body has insufficient iron for normal function, is clinically characterized by a decrease in serum iron and transferrin saturation (TSAT) and an increase in soluble transferrin receptor (sTfR). Iron deficiency can progress to iron deficiency anemia (IDA), characterized by compromised red blood cell synthesis and a reduction in hemoglobin (Hb) concentration [66].

Brain iron deficiency during development is associated with memory and learning impairments. Strong evidence exists that the hippocampus is particularly affected by iron deficiency during development. Perinatal iron deficiency in the developing rat brain reduces neuronal metabolic activity in the hippocampus [67]. Early-life ID has also been shown to alter hippocampal glucose transport by increasing glucose transporter-1 (GLUT1) expression [68]. Rats placed on an iron-deficient diet at an early age had deficits in passive avoidance, active avoidance, hippocampus-dependent trace fear conditioning, and Morris Water Maze performance [69,70,71,72]. A particularly unfortunate aspect of early ID is the persistence of cognitive impairments even after iron supplementation. Pre-natal iron-deficient mice pups had deficits in reference memory in a radial arm maze even after receiving sufficient iron after birth. The cognitive deficits coincided with reduced neurogenesis and brain-derived neurotrophic factor (BDNF) in the hippocampus [73]. Another group used whole-genome bisulfite sequencing to identify differentially methylated loci in postnatal day 15 ID rat hippocampus. They identified changes in DNA methylation in networks regulating neuronal development, axonal guidance, cAMP-mediated signaling, and reelin signaling. They concluded that DNA methylation could be an important epigenetic mechanism leading to hippocampal gene dysregulation in early ID, which could be a contributing factor for persistent symptoms post iron supplementation [74]. Similar long-term effects are seen in children born with ID who continue to perform poorly on neurocognitive tests [75,76,77,78]. Iron deficiency in adulthood is associated with depression and difficulty completing complex cognitive tasks [79].

### 5.2. Iron Deficiency and Monoamines, ADHD, and RLS

Tyrosine hydroxylase and tryptophan hydroxylase are both iron-dependent enzymes and are the rate-limiting steps of catecholamine and serotonin/melatonin synthesis pathways, respectively. Thus, the consequences of ID have long been thought to at least in part be due to changes in these neurotransmitter systems [79]. These are often difficult to study directly because of regional brain iron metabolism regulation and the use of correlative instead of mechanistic studies relying mostly on systemic iron or ferritin concentrations as markers of ID.

This is illustrated in a 2017 study in which C57BL/6 mice after one month of an iron-restricted (IR) diet had decreased ferritin levels in the liver and hippocampus but increased ferritin levels in the striatum. The striatum also had a decrease in the level of dopamine metabolites 3,4-dihydroxyphenylactic acid (DOPAC) and homovanillic acid (HVA), but the prefrontal cortex had an increase in HVA and enhanced monoamine oxidase (MAO) activity, while no changes were seen in the hippocampus [80]. A 2022 study by the same group demonstrated that rats fed an iron-restricted diet and control rats had similar levels of iron and ferritin in the striatum and hippocampus. In the striatum, however, metabolic enzymes involved in glycolysis were reduced while the level of lipid catabolism and TCA cycle enzymes increased, suggesting a metabolic shift to β-oxidation, which is more prone to ROS production. In the hippocampus, the IR diet led to an increase in chaperone proteins and a reduction in PARK7 and α-synuclein, suggesting an unhealthy environment with an increase in oxidative stress [81]. This highlights the possibility of ID negatively impacting the brain without changes in the markers we associate with iron metabolism.

Attention-deficit/hyperactivity disorder (ADHD) is estimated to have a worldwide prevalence of 5.29% in children and between 2.5% and 4.4% in adults. Changes in multiple neurotransmitter systems involved in executive function, working memory, emotional regulation, and reward processing have been implicated in ADHD including dopamine (DA), norepinephrine (NE), serotonin (5-HT), acetylcholine (ACH), opioid, and glutamate (GLU) pathways. First-line pharmacotherapies are psychostimulants including amphetamines and methylphenidate, which act to increase central DA and NE activity in the cortex and striatum [82].

Iron deficiency is thought to contribute to ADHD in a few ways. As a cofactor of tyrosine hydroxylase, the rate limiting step in dopamine synthesis, a deficiency in iron could reduce dopamine synthesis. Iron deficiency is also associated with a decrease in dopamine transporter activity, increased extracellular dopamine, and reduced dopamine receptors in the striatum [83].

Correlative studies, however, have conflicting reports of an association between iron deficiency and ADHD. A 2017 meta-analysis found lower serum ferritin levels in patients with ADHD than in healthy controls [83]. A 2018 meta-analysis study looked at research investigating the relationship between peripheral iron levels and ADHD and found a significant association between lower serum ferritin levels in ADHD children, but no significant difference in serum iron or transferrin levels. The severity of ADHD also corelated with ID [84]. In 2019, a South African study of 245 outpatient children and adolescents found no significant correlation between ADHD and iron deficiency [85]. Meanwhile, another study that same year demonstrated an increase in serum hepcidin levels in medication-naïve ADHD patients, possibly contributing to iron dysregulation in the brain [86]. Medication-naïve children also had reduced brain iron compared to controls and psychostimulant-medicated patients in a separate study. Long-term psychostimulant use may help compensate in ADHD children for a decrease in age-related basal ganglia iron accumulation that typically occurs during development [87]. In terms of treatment, a 2021 systemic review of iron and zinc supplementation treatment in ADHD children and adolescents found that low zinc and iron levels correlated with ADHD severity and worse treatment outcomes. In the nine studies that met their criteria, zinc and iron supplements were associated with improvements in ADHD severity though the effect size was low and limited to specific ADHD symptoms/measures. They also suggested that zinc and iron supplementation may be particularly useful for specific subgroups of patients [88,89].

Restless leg syndrome (RLS) is a sensorimotor neurological disorder characterized by a compulsion to move the legs and a sense of restlessness particularly at night. Common comorbid conditions include idiopathic pulmonary fibrosis, end-stage renal disease, irritable bowel syndrome, ADHD, and insomnia. The “iron–dopamine” hypothesis has been put forward as a possible cause for RLS and involves regional brain iron deficiency, particularly in the substantia nigra, disturbing dopamine neurotransmission [90].

The nigrostriatal dopamine system plays an important role in RLS. ID in mice was shown to affect gene expression in the ventral midbrain including increasing expression of hemoglobin, beta adult chain (Hbb-b1), chemokine (C-X-C motif) ligand 12 (Cxc112), TFRC, Alas2, and radical S-adenosyl methionine domain containing 2 (Rsad2) [91].

A 2019 study looked at the incidence of ADHD and obsessive-compulsive disorder (OCD) in RLS patients. The prevalence of patients with RLS with ADHD symptoms was 27.62%; with OCD symptoms, 7.62%; and with both, 7.62%, which is higher than the estimated prevalence in the general population. They did not, however, observe a significant association between the presence of ADHD or OCD symptoms in RLS patients and iron or ferritin serum concentrations, but as these are not necessary indicators of brain iron stores, brain imaging iron quantification is necessary to rule out an association between iron and RLS patients with ADHD and/or OCD [92].

### 5.3. Iron and Anxiety

The relationship between iron and anxiety is controversial. Ceruloplasmin-knockout mice develop an anxiety phenotype at 3 months of age that is associated with reduced iron levels in the hippocampus, anxiety-like behavior in open field and elevated plus tests, elevated plasma corticosterone, and a reduction of serotonin (5HT), norepinephrine (NE), and brain-derived neurotrophic factor (BDNF) in the hippocampus. No changes in learning and memory were observed [93]. Older CpKO mice (16 months) exhibit increased iron deposition in the cerebellum and brainstem regions, loss of dopaminergic neurons in the brainstem, and motor deficits [94]. Aceruloplasminemia in humans can present with cognitive decline, depression, anxiety, and behavioral changes in addition to the classic phenotype of ataxia and hyperkinetic movement disorders [95,96].

Recently, a link between iron transport and anxiety was reported. Two unidirectional pathways were identified for iron transport in the brain. The first is a pathway from the ventral hippocampus (vHip)–medial prefrontal cortex (mPFC)–substantia nigra (SN) and the second is from the thalamus–amygdala–mPFC. Iron transport in these pathways was dependent on neuronal activity. Interestingly, interruptions in the first pathway but not the second were associated with anxiety-like behaviors. The study also revealed an elaborate method of iron regulation in the mPFC. The mPFC under low-iron conditions will take up iron from the vHip and amygdala (even to the point of causing those regions to become iron deficient) and push excess iron to the SN, which does not export it, likely leading to iron accumulation in the SN over time [97].

## 6. Irp2-Knockout Mouse Models

### 6.1. The Rouault Irp2 KO Model

**Figure 1:**
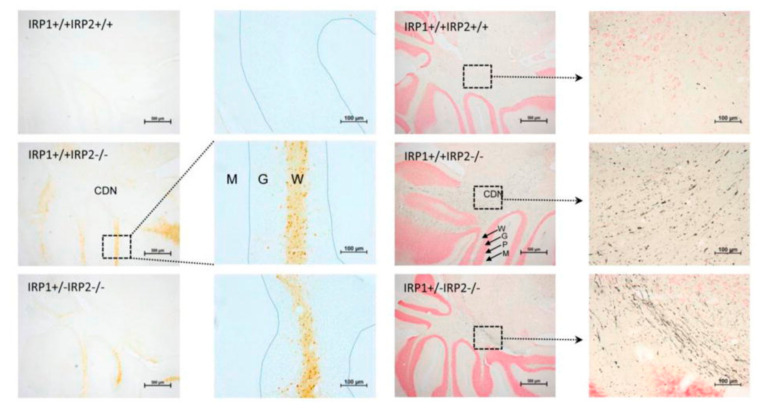
Perls’ DAB stain (**left**) and amino cupric silver stain (**right**) of WT (**top**), Irp2^−/−^ (**middle**), and Irp1^+/−^Irp2^−/−^ (**bottom**) mouse cerebellum sections showing a dose-dependent effect of IRP ablation on iron accumulation in white matter (yellow) and axon degeneration (black strands). Abbreviations: CDN—cerebellar deep nuclei, W—white matter, G—granular cell layer, P—Purkinje cell layer, M—molecular layer. Modified with permission from ref. [1] 2015 Elsevier.

To investigate iron metabolism misregulation, the Rouault lab created the first IRP2 KO mouse model by inserting a PGK-neomycin gene into exon 3/4 of the Irp2 gene. After six months of age, the IRP2-null mice developed motor deficits characterized by abnormal gait; ataxia; kyphosis; tremor; postural abnormalities; and poor performance on hanging wire, balance, and grip strength tests. Ferric iron stains revealed iron accumulation in cerebellar white matter and deep nuclei, caudate putamen, thalamus, and colliculi. Axonal degeneration was detected in white matter tracts colocalizing with sites of iron accumulation (Figure 1).

The iron accumulation appeared to precede the axonal degeneration based on iron stains of young mice. Ferritin overexpression indicated that the accumulated iron was sequestered and unavailable for use by iron proteins [98]. It was later determined that axonal degeneration also occurred in the lower motor neurons in the spinal cord based on the accumulation of myelin-dense bodies (MDBs). Upper motor neurons also had morphological abnormalities and indicators of chromatolysis. Additionally, Irp2^−/−^ mice bred to have only one copy of IRP1 (Irp1^+/−^ Irp2^−/−^) had a more severe phenotype, indicative of a dose-dependent effect [99]. Treatment with Tempol, a stable nitroxide, increased IRP1 IRE-binding activity and partially restored iron homeostasis, indicating that iron regulatory proteins can compensate for each other [100].

The mitochondrial respiratory chain complexes require iron–sulfur clusters and heme cofactors to function, and defects in their functionality have severe consequences. In spinal cord tissue from Irp2^−/−^ mice, the activity of complexes I, II, and III were significantly decreased without a concomitant decrease of component proteins. Because complex IV, which utilizes a heme cofactor, was unaffected, the loss of activity was likely due to a loss of iron–sulfur cluster biogenesis, which is used by complexes I–III. Electron microscopy also revealed swollen mitochondria with disrupted and vacuolized cristae [62]. Neurons are particularly dependent on their mitochondria due to the amount of energy required to power ion pumps and maintain the neuron’s resting membrane potential. Therefore, a decrease in iron–sulfur biogenesis and, thus, mitochondrial function could result in neurodegeneration.

The dopamine (DA) system in these Irp2^−/−^ mice was investigated using harvested CNS tissue from 16–19-month-old mice. Tyrosine hydroxylase (TH) was decreased 20–25% in both the dorsal and ventral striatum, while in the ventral striatum, approximately 40% of dopamine transporter (DAT) and vesicular monoamine transporter (VMAT2) was lost compared to WT mice. DA decreased by 20% in the dorsal striatum with also a 30% increase in DA turnover. The researchers concluded that loss of Irp2 in these mice and the resulting iron misregulation led to changes in DA regulation in the striatum [101].

It has also been demonstrated that impaired Fe–S cluster biogenesis in b cells of Irp2^−/−^ mice leads to impaired proinsulin processing, reduced insulin content and secretion, and the development of diabetes in these mice [102].

### 6.2. The Hentze Irp2 KO Model

The Hentze group created a second Irp2^−/−^ mouse model using a gene trap construct and backcrossing with C57Bl/6J mice [103]. At 13–14 months of age, the researchers did not observe ataxia, tremor, bradykinesia, and postural abnormalities as were seen in the Rouault model. The Irp2^−/−^ mice on an accelerating rotarod test had a two-fold reduction in latency to fall, but no effect was observed in a forelimb grip strength test. The mice had a similar increase in ferritin and decrease in TFR1 expression in the brain, though total non-heme iron was unchanged, and no iron deposits were observed after 3,3′-diaminobenzidine (DAB)-enhanced Perl’s staining of serial brain sections. Calbindin staining of cerebellar Purkinje cells showed intact dendritic trees and axons with no cell loss. Electron microscopy also showed no changes in mitochondrial morphology. Interestingly, the mice showed a mild tendency for increased horizontal locomotion on a modified hole board test [104].

### 6.3. The Leibold Irp2 KO Model

A third model was then created in 2014 by the Leibold group using a self-excision cassette with neomycin linked to Cre-recombinase inserted into exon 3 of IREB2 and backcrossing with C57Bl/6J mice [105]. Irp2^−/−^ mice between 45 and 63 weeks of age did not show tremors, kyphosis, or abnormal gait. Both rotarod performance and nociceptive heat tolerance (measured using a hot-plate assay at 52 °C) were impaired. These Irp2^−/−^ mice showed reduced locomotion, speed of movement, and vertical exploratory activities in a modified hole board test. Similar to the Rouault and Hentze models, there was no significant difference in total brain iron content. Iron deposition was observed in the cortex, thalamus, and cerebellum; and iron accumulation in axonal tracts, cerebellar white matter, and oligodendrocytes, matching the Rouault group. Iron was reduced in Purkinje neurons and in CA1 pyramidal neurons. The Leibold group concluded that increased expression of ferritin and decreased expression of TfR1 led to iron sequestration and a decrease in iron import, respectively, causing functional iron deficiency in neurons [105].

### 6.4. Additional Mechanistic Studies

Mouse embryonic fibroblasts derived from the Rouault Irp2^−/−^ model were used to examine changes in metabolism [106]. Their studies revealed that mitochondrial dysfunction in Irp2^−/−^ cells leads to a metabolic shift between OXPHOS to aerobic glycolysis.

Surprisingly, the ATP content increased in Irp2^−/−^ MEF cells. The transcription factors Hif1a and Hif2a were upregulated. As Hif1a and Hif2a are regulated by oxygen and iron, this upregulation could be a result of inactivation of iron-dependent prolyl hydroxylases needed to mark HIF proteins for degradation due to functional iron deficiency, and in vivo by induced anemia in addition to the regulation of Hif2a by IRP/IRE binding [107,108]. The glycolysis proteins hexokinase 2 (HK2), glucose transporter 1 (Glut1), and lactate dehydrogenase A/B (LdhA/B) were significantly increased. Oxygen consumption rate (OCR) and extracellular acidification rate (ECAR) were measured using an Agilent Seahorse Analyzer. OCR is an indicator of mitochondrial respiration, and Irp2^−/−^ cells had a lower resting OCR and maximal mitochondrial capacity after treatment with carbonyl cyanide p-(trifluoromethoxy)phenylhydrazone (FCCP) than WT cells. Conversely, Irp2^−/−^ cells had a much higher ECAR, an indicator of glycolytic rate, after glucose and oligomycin treatment. Specific inhibitors for Hif1a (PX-478) and Hif2a (PT-2385) were used to investigate the mechanism for this switch. Inhibition of Hif2a increased the expression of iron-related and mitochondrion-related proteins (Pdh, Ndufs1, SdhB, Uqcrfs1, Fxn, and IscU). Inhibition of Hif1a reduced the expression of LdhA and HK2. Additionally, basal respiration, maximal respiration, and OXPHOS-dependent ATP production increased after Hif2a inhibition, while both glycolysis and glycolysis capacity were suppressed after Hif1a inhibition. The activities of mitochondrial respiratory complexes I, II, and III also increased after inhibition of Hif2a. The study concluded that loss of Irp2 induces a metabolic switch to glycolysis due to an increase in the levels of Hif1a and Hif2a [106].

This metabolic switch was then examined in vivo. Again, increased ferritin and decreased TfR1 levels were observed in the cerebrum, cerebellum, brainstem, and spinal cord tissues of Irp2^−/−^ mice. There was no difference in expression of Hif1a, but glycolysis-related proteins (ldhA, Glut1, and Hk2) were upregulated. Hif2a levels were around 50% higher, while the levels of IscU and Fxn were 50–70% of normal. Subunits of mitochondrial respiratory complexes I (Ndufs1), II (SdhB), and III were also about half the level observed in WT mice. Treatment with the Hif2a inhibitor PX-2385 rescued rotarod and hang test performance as well as the anemia of Irp2^−/−^ mice. The inhibitor also suppressed degeneration of cerebellar Purkinje cells observed in H&E staining. The treatment also reduced mitochondrial dysmorphology in the spinal cord. The activity of complex I and II was also restored. These effects were not observed with the Hif1a inhibitor treatment [109]. This study implicates Hif2a in the Irp2^−/−^ neurodegenerative disease mechanism as the cause of the energy metabolism shift from glycolysis to OXPHOS in vivo, and Hif2a may be a potential therapeutic target in the future. Interestingly, Irp1^−/−^ mice develop polycythemia and pulmonary hypertension due to an increase in Hif2a, and this can also be reversed with a Hif2a inhibitor (MK-6482) [110,111].

On the opposite side of IRP2-related pathology, loss of FBXL5 is embryologically lethal. FBXL5^−/−^ embryos appear normal until E8.5, when they begin to undergo resorption, manifesting growth retardation and massive hemorrhage. Embryonic lethality is thought to be due to aberrant IRP activity leading to iron accumulation and oxidative stress. Ablation of IRP2 rescues FBXL5^−/−^ embryos from death, and these mice develop normally. Iron metabolism markers in Fbxl5^−/−^Irp2^−/−^ were nearly identical to Irp2^−/−^ apart from an increase in serum iron concentration and transferrin saturation [13].

## 7. The Discovery of the First IRP2-Null Patients

Recently, clinical exome sequencing at the Neurogenetics Clinic at the Hospital for Sick Children in Toronto, Canada, has identified a 16-year-old male patient with bi-allelic loss-of-function mutations in IREB2 resulting in the absence of the IRP2 protein. The patient was diagnosed with dystonic cerebral palsy by age one. Brain MRIs revealed extra-axial CSF spaces and prominent ventricles indicative of cerebral volume loss. The frontal regions also showed delayed myelin maturation and moderate loss of white matter. The patient also developed a severe movement disorder and generalized tonic–clonic seizures by the age of nine. By age 12, brain MRI indicated progressive cerebral atrophy and white matter volume loss. The patient also had microcytic hypochromic anemia unresponsive to iron supplementation (hemoglobin: 11.4 g/dL; mean corpuscular volume: 65.6 fl; serum ferritin: 225.5 mg/L; and zinc protoporphyrin: 352.0 mmol/mol Hgb; normal serum transferrin and iron) and severe developmental delays including never learning to walk or speak [112].

Immortalized lymphoblastoid cell lines from the patient and his father (control) were generated at the Centre for Applied Genomics in Toronto, Canada. The patient lymphoblasts had reduced expression of TfR1 and increased expression of ferritin indicating iron misregulation. An increase in IRP1-IRE binding activity partially compensated for the loss of IRP2 protein. The cell lines also had decreased activities in complexes I, II, and IV as well as a decrease in the labile iron pool, even though total cellular iron levels were normal. These results match the Irp2^−/−^ mouse data and support the hypothesis that loss of IRP2 causes iron metabolism misregulation resulting in functional iron deficiency [112].

Once the case study of this patient was published, a second group in Australia came forward with information on a second male patient with bi-allelic variants in IREB2. The patient died at the age of 10 years old in January 2019 due to progressive neurological disease. The patient presented with hearing loss, poor visual fixation, feeding problems, plagiocephaly, and hypotonia by 3 months of age. The patient was also diagnosed with cerebral palsy. He developed the ability to recognize faces and make brief facial expressions as well as reach for toys. At first, he could use a walker for movement, but he lost mobility over time due to mild dystonia and athetosis. MRIs at 5 and 24 months of age showed a thickened corpus callosum and progressive volume loss in the frontal lobes.

An EEG was slightly abnormal, but the patient did not have clinical seizures. The patient sometimes had mild microcytic anemia with an average hemoglobin level of 12.0 g/dL, elevated serum ferritin (peak at 449 mg/L), and normal to low serum transferrin and iron levels. The patient died from fungal sepsis and malnutrition from an inability to tolerate gastric feeding tubes. Trio exome sequencing revealed that the patient was a compound heterozygote for two variants in IREB2: a maternally inherited missense variant and a paternally inherited in-frame deletion [113].

A third patient was referred to the Mitochondrial Medicine Clinic at the Seattle Children’s Hospital to be evaluated for suspected mitochondrial disease. Circumference of the head was low at 34.5 cm. By 5 months, the patient had developed hypotonia, decreased movements, and a viral illness complicated by nystagmoid eye movements. Optic nerve dysfunction was also observed in an eye exam. Additionally, visual evoked potentials revealed cortical visual dysfunction, and social smile and vision fixation were missing. The patient developed increasing quick jerks in his extremities around 8 months. At 11 months, similarly to the first patient, he developed epileptic spasms and more subtle seizures characterized by eye rolling and neck extension that responded to antiseizure medication. An EEG demonstrated background diffuse slowing and multifocal epileptiform discharges over both hemispheres, and when repeated at 5 years of age, demonstrated slow background with bilateral epileptiform discharges from the temporal region. MRI did not reveal structural abnormalities.

The male, 7-year-old patient was born to non-consanguineous parents by C-section at 36-1/7 weeks of gestation due to a failure to progress. The patient currently presents with progressive postnatal microcephaly (OFC −2.6 SD) and small stature. He is severely developmentally impaired. He cannot roll or sit on his own or lift his head upright when in a sitting position. He is hypotonic in his core and modestly hypertonic in his extremities. Spontaneous movement is decreased, and he displays stereopathies of his hands. He knows about 10 words but is unable to string them into sentences. He is borderline anemic (hemoglobin 11.7 g/dL; serum iron 25 mmol/L; and serum ferritin 12 ng/mL). Analysis of mitochondrial respiratory complex activity in a muscle biopsy showed impaired mitochondrial function (complex I: 28% of normal; complex II: 52% of normal).

Clinical exome sequencing identified compound heterozygous missense mutations in IREB2 leading to effectively undetectable levels of IRP2 protein, upregulation of ferritin, and downregulation of TfR1 in patient lymphoblasts. Treatment with a proteasomal inhibitor MG-132 slightly increased IRP2 protein levels, but expression of ferritin heavy (FTH) and light (FTL) chains or TfR1 were not restored to normal, indicating that the missense mutations impeded IRP2 function in addition to causing the protein to misfold or become unstable [114].

The identification of three patients over a short period of time with highly similar presentations indicates that biallelic variants in IREB2 may cause a new subclass of neurodegenerative disorders (Table 1). It is likely that, as access to sequencing increases, more patients will be identified. The third patient in particular expands the scope of IRP2-related disorders by demonstrating that severe disease can result from single-nucleotide mutations that could cause missense mutations or abnormal splicing. Other IREB2 variants that affect the function of IRP2 may cause more-mild or less-obvious neurological symptoms. Further research is, therefore, needed to fully understand the possible spectrum of disease manifestations. The neurodegenerative phenotype of the Irp2^−/−^ mouse model is milder than the documented human disease [98]. This model may, therefore, be useful in discovering less-severe neurological symptoms that could present in human patients.

## Figures and Tables

**Table 1:** Summary of IRP2-null patients’ symptoms.

Patient	Costain et al., *Brain* (2019) [112]	Cooper et al., *Brain* (2019) [113]	Maio et al., *Brain Communications* (2022) [114]
IREB2 mutation (GenBank: NM_004136.2)	c.1069G > T; c.1255C > T	c.2353G > A; c.1329_1331del	c.2240G > A; c.656A > C
Amino acid substitutions in IRP2	p.G357X; p.R419X	p. G785R; p.S444del	p.G747E; p.E219A
Cerebral palsy	+	+	
Cerebral volume loss on MRI	+	+	−
Movement disorder	+	+	+
Seizures	+	−	+
Microcytic anemia (hemoglobin)	+(11.4 g/dL)	+(12.0 g/dL)	+(11.7 g/dL)
Reduction of transferrin/TFRC	+	+	+
Increase in serum ferritin	+(225.5 µg/L)	+(449 µg/L)	−(12 µg/L)
Mitochondrial dysfunction	+		+
Visual symptoms	+	+	+
Developmental delays	+	+	+

## Data Availability

Not applicable.
